# It’s not just snot! Mucus: Nature’s Multifaceted Secretion

**DOI:** 10.1038/s44319-026-00729-0

**Published:** 2026-03-13

**Authors:** Corey A Stevens, Katharina Ribbeck

**Affiliations:** 1 Department of Biological Engineering, Massachusetts Institute of Technology, Cambridge, MA 02139 USA; 2 Department of Biological Engineering, Koch Institute for Integrative Cancer Research, Massachusetts Institute of Technology, Cambridge, MA 02139 USA

**Keywords:** Digestive System, Immunology, Respiratory System

## Abstract

Unfairly labeled as ‘snot’ or ‘phlegm’ mucus is a biological marvel that plays a crucial role in protecting the body from pathogens and pollutants. Its multiple functions and extraordinary properties have inspired new biomaterials.

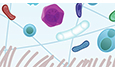

Few bodily substances evoke such strong reactions as mucus. For most of us, our experiences with mucus are intimate and often unpleasant, usually involving a quick disposal. Terms like “snot” or “phlegm” are synonymous with illness or embarrassment. Yet, this ubiquitous secretion is a biological marvel, protecting an estimated 185 m^2^ of internal surface area—hydrating, lubricating, and safeguarding delicate tissues within the oral, respiratory, digestive, ocular, and female reproductive tracts (Johansson and Hansson, 2016) (Fig. 1).

**Figure 1:**
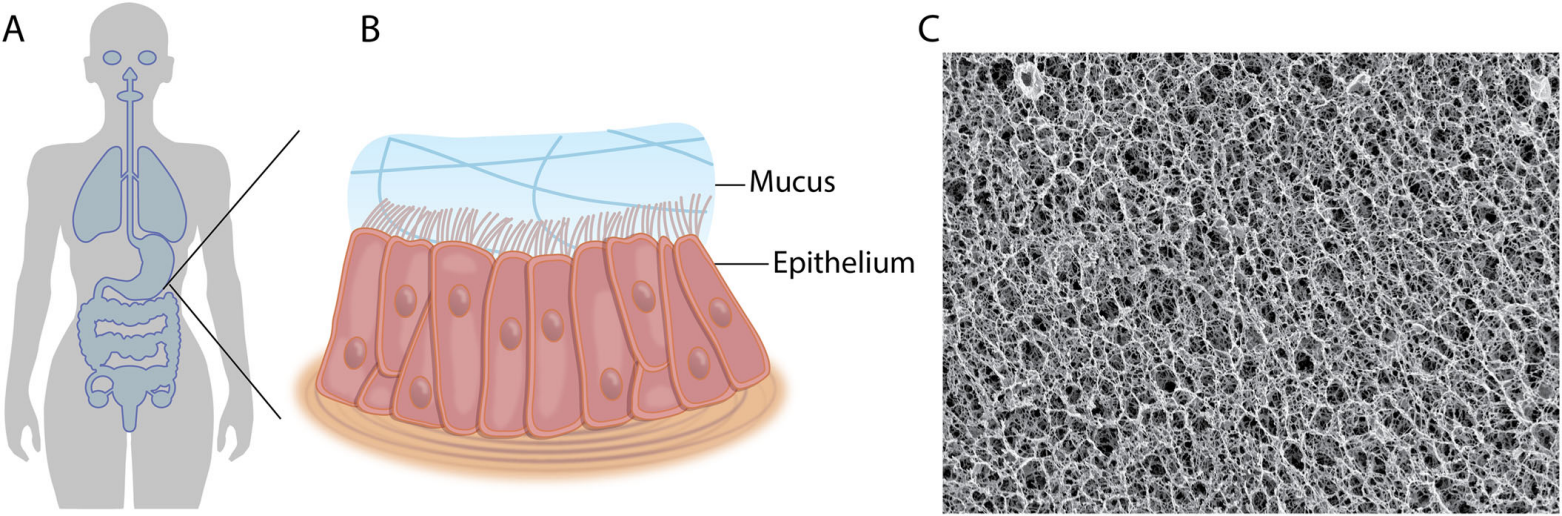
The mucus barrier is our first line of defense. (**A**) Mucus lines and protects all exposed internal surfaces, including the respiratory, gastrointestinal, and reproductive tracts. (**B**) At the tissue level, mucus overlays epithelial cells and is propelled by cilia to clear trapped particles. (**C**) Structurally, mucus is a cross-linked network of mucin polymers that forms a selective, gel-like mesh. This unique architecture allows mucus to serve as both a physical filter and a biochemical shield against pathogens and environmental insults.

“… this ubiquitous secretion is a biological marvel, protecting an estimated 185 m^2^ of internal surface area…”

Comprised primarily of water, mucus is a sophisticated gel infused with specialized, sugar-coated proteins called mucins with a supporting cast of lipids, salts, and potent antimicrobial molecules such as lysozymes and defensins (Thornton and Sheehan, 2004). These essential components are produced by specialized cells, including “goblet cells” in the eyes, lungs, and gut, as well as additional dedicated glands in other tissues (Hansson, 2012).

Our fascination—and revulsion—with mucus isn’t new. In Hippocratic and Galenic medicine, phlegm was considered one of the four essential bodily “humors” alongside blood, yellow bile, and black bile (Nutton, 2005). An excess or deficiency of phlegm was believed to dictate temperament and cause disease, giving rise to terms like “phlegmatic” to describe a calm or sluggish disposition. Today, mucus is still one of the original biomarkers: clear mucus signals wellness, whereas a shift to green or yellow often indicates an infection of the airways. Yet, our interpretation of mucus has changed dramatically, with the focus shifting from its presence as a general humor to its crucial role in preventing infection.

Mucus-like substances play intriguing roles throughout the animal kingdom and even in pop culture. Think of a mother animal meticulously licking her offspring: the mucin-rich saliva aids in wound healing and prevents infection. We instinctively do something similar when we “lick our wounds” after a minor cut, leveraging saliva’s inherent healing properties. And what child hasn’t been fascinated by “boogers” or the gooey ectoplasm in *Ghostbusters* or the mutagenic ooze of *Teenage Mutant Ninja Turtles*? These examples highlight mucus’s pervasive, often humorous, and surprisingly versatile presence. As we peel back the layers of it multiple roles, it becomes clear that this often-misunderstood substance plays a key role in health. Delving deeper into its multifaceted functions not only reveals the genius of mucus biology but also opens the door to novel medical breakthroughs.

“And what child hasn’t been fascinated by “boogers” or the gooey ectoplasm in *Ghostbusters* or the mutagenic ooze of *Teenage Mutant Ninja Turtles*?”

## The body’s invisible wall

Mucus serves as a living barrier, evolved to guard our most vulnerable internal pathways by trapping harmful invaders like viruses, bacteria, and pollutants and preventing them from entering the body. Modern science has revealed that this defense relies on two ingenious mechanisms: size-based filtering and sticky binding interactions.

**Figure 2:**
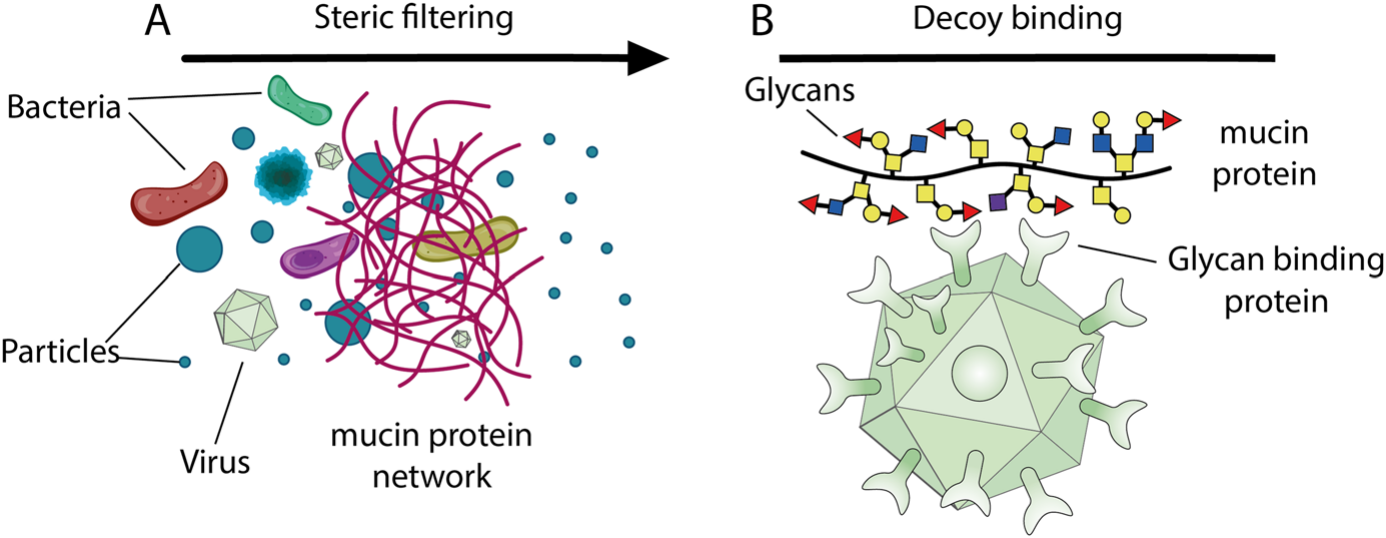
Mucus protects vulnerable internal surfaces through two primary and complementary mechanisms. (**A**) Size-based filtering: a dense mucin “micro-net” with nanoscale-to-microscale pores traps larger invaders such as viruses, bacteria, and pollutants, which are then cleared by mucociliary transport. (**B**) Sticky binding: mucin glycans act as molecular “handcuffs” and “decoys,” binding particles and mimicking host-cell receptors to immobilize pathogens. Together, these physical and biochemical defenses prevent harmful agents from reaching underlying tissues.

Mucus forms a dense, cross-linked network of mucin glycoproteins with pores ranging from 100 nanometers to several micrometers (Lai et al, 2009). This “mucus micro-net” is a highly selective sieve (Fig. 2A). Tiny particles and smaller molecules can slip through relatively easily, but larger ones—like many viruses, bacteria, or airborne pollutants—get stuck within this sticky matrix. Once trapped, mucociliary clearance, a sweeping motion of tiny hairs, or cilia, in the airways that constantly propels the mucus upwards, efficiently removes these captured particles, preventing them from reaching or damaging vulnerable cells (Button et al, 2018).

Mucins are rich in negatively charged sugar molecules, particularly sialic acid and sulfate groups, providing numerous potential binding sites to airborne particles (Takagi et al, 2022) (Fig. 2B). Positively charged molecules, peptides, and even some therapeutics can become electrostatically bound or ‘handcuffed’ to mucins. While this stickiness can complicate drug delivery by inhibiting therapeutic agents from *crossing* the mucus barrier, scientists are leveraging this feature by designing “muco-adhesive” drugs that are *meant* to stick to mucus for a controlled, sustained release. Furthermore, the sugar chains on mucins act as “decoys,” mimicking the docking sites that viruses and bacteria normally use to attach to cells (Kaler et al, 2022). For instance, the influenza virus tries to clear its path by cutting off sialic acid from mucin glycans. But by binding to these decoy glycans, it becomes immobilized and is eventually cleared from the airways before it can reach its target cells.

This sophisticated interplay of physical trapping and molecular binding highlights the incredible versatility of mucus in shielding the body from environmental hazards and infections. Continued research into the properties of mucus not only deepens our biological understanding of this protective secretion but also paves the way for exciting advancements in medicine and environmental protection.

“This sophisticated interplay of physical trapping and molecular binding highlights the incredible versatility of mucus in shielding the body from environmental hazards and infections.”

## Life within the gel: the mucus microbiome

**Figure 3:**
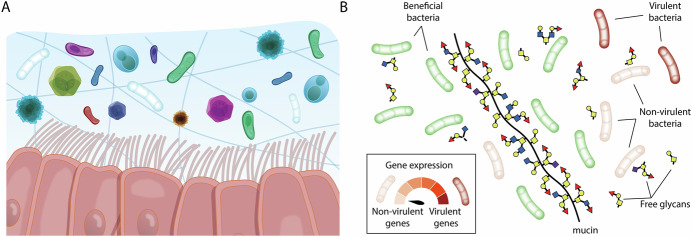
Mucus is home to much of the human microbiome, selectively hosting beneficial microbes. (**A**) Schematic illustration of the mucus barrier as a structured gel above epithelial cells. Diverse microbes inhabit the mucus layer, where beneficial bacteria are supported by access to glycans. (**B**) Commensals (green) are shown thriving on mucin-derived glycans, while pathogens (red) exhibit reduced virulence, represented by the dial-down effect (light red). This behavior highlights the dual role of mucus in supporting symbionts while restraining potential pathogens.

Far from being sterile, mucus is a dynamic ecosystem that hosts the vast majority of the body’s microbiome (Johansson and Hansson, 2016) (Fig. 3A). This diverse community of bacteria, viruses, fungi, and other microorganisms is neither a random collection of organisms nor an army of invaders; these microbes are often essential in maintaining health. For instance, the mucus layer in the gut provides a home for beneficial bacteria that help digest food and produce essential vitamins.

How does mucus host a thriving, mostly beneficial microbial population without constantly triggering the immune system? It’s a marvel of biological pacification, with mucin glycans serving as both selective food and anchor points (Tailford et al, 2015). First, these carbohydrates have a unique structure, requiring specific enzymes for microbes to break them down. Consequently, these glycans can be used only by certain microbes as a food source, effectively selecting for a specialized group of beneficial bacteria. Second, glycans function as anchor points, providing a preferred spot for host-supportive microbes to reside. These anchor points encourage the formation of a healthy, balanced microbial community.

Crucially, when problematic pathogens encounter mucus, the interaction is often not about outright destruction. Instead of killing pathogens, mucin glycoproteins can tame them by downregulating the expression of virulence genes and inhibiting cellular adhesion (Wheeler et al, 2019) (Fig. 3B). Although mucus contains antimicrobial peptides and enzymes that *can* selectively destroy harmful microorganisms, the “taming” aspect of mucus is a distinct and vital mechanism.

“Although mucus contains antimicrobial peptides and enzymes that *can* selectively destroy harmful microorganisms, the “taming” aspect of mucus is a distinct and vital mechanism.”

Despite significant progress in understanding the role of mucus in the human microbiome, many questions remain unanswered. Scientists are still unraveling the mechanisms by which mucus orchestrates a healthy balance of these microbial communities. Researchers are also investigating how changes in mucus composition and properties—likely influenced by diet, lifestyle, or environmental factors—can lead to dysbiosis, whereby harmful microbes gain an upper hand. This imbalance is linked to serious conditions such as inflammatory bowel disease (IBD) and chronic sinusitis. Understanding this intricate “life within the gel” is crucial for developing new strategies to manipulate this environment, which can lead to novel treatments for microbiome-targeted therapies.

## The architects and guardians of mucus

Epithelial cells, which form the linings of mucosal surfaces, are the primary architects of mucus by producing and secreting mucin proteins. But the role of these cells extends beyond production; they constantly interact with mucus to sense and respond to changes. For example, the dynamic process of mucociliary clearance, where cilia rhythmically sweep mucus and trapped particles away from sensitive surfaces, was first studied in the mid-20th century, revealing the active nature of this host defense.

Consider the common misery of hay fever to illustrate the crucial role of mucus. When pollen hits the mucous membranes of the nose and eyes, it triggers an allergic reaction. This reaction often leads to increased mucus production (runny nose) and changes in consistency, with mucus becoming more watery, to flush out the irritant. Conversely, in cold weather, mucus thickens, potentially compromising its defense against respiratory viruses, as a thicker mucus might not clear pathogens as effectively.

Another exciting area of research explores how host immune cells function within mucus. In vital organs such as the eyes and lungs, innate immune cells, such as neutrophils, are crucial defenders against invading pathogens. Yet, how the surrounding mucus influences these cells is still poorly understood. Recent findings suggest that thick, viscous gels might enhance immune cell motility, raising intriguing questions about mucus’s role in guiding these defensive cells to their targets.

Mucus dysfunction is a central factor in diseases such as asthma, IBD, and cystic fibrosis (CF), where dying immune cells release large amounts of DNA and other sticky filaments, which increases mucus thickness and stickiness (Fahy and Dickey, 2010). In CF, for example, this results in abnormally thick, dehydrated mucus that fosters chronic infections and inflammation. In IBD, breaches in the mucus layer allow commensal microbes to trigger harmful immune responses. Unraveling how immune cells are influenced by mucosal changes therefore holds immense potential to inform new treatments for mucus-associated diseases.

By way of another example, cervical mucus in the reproductive system serves as both a barrier and a dynamic gatekeeper. It changes viscosity across the menstrual cycle to either block or facilitate sperm transport. At ovulation, the mucus forms channels that facilitate sperm motility and survival, biochemically cross-linking with sperm to protect and direct them towards the egg to increase the chance of fertilization.

## A widespread phenomenon

**Figure 4:**
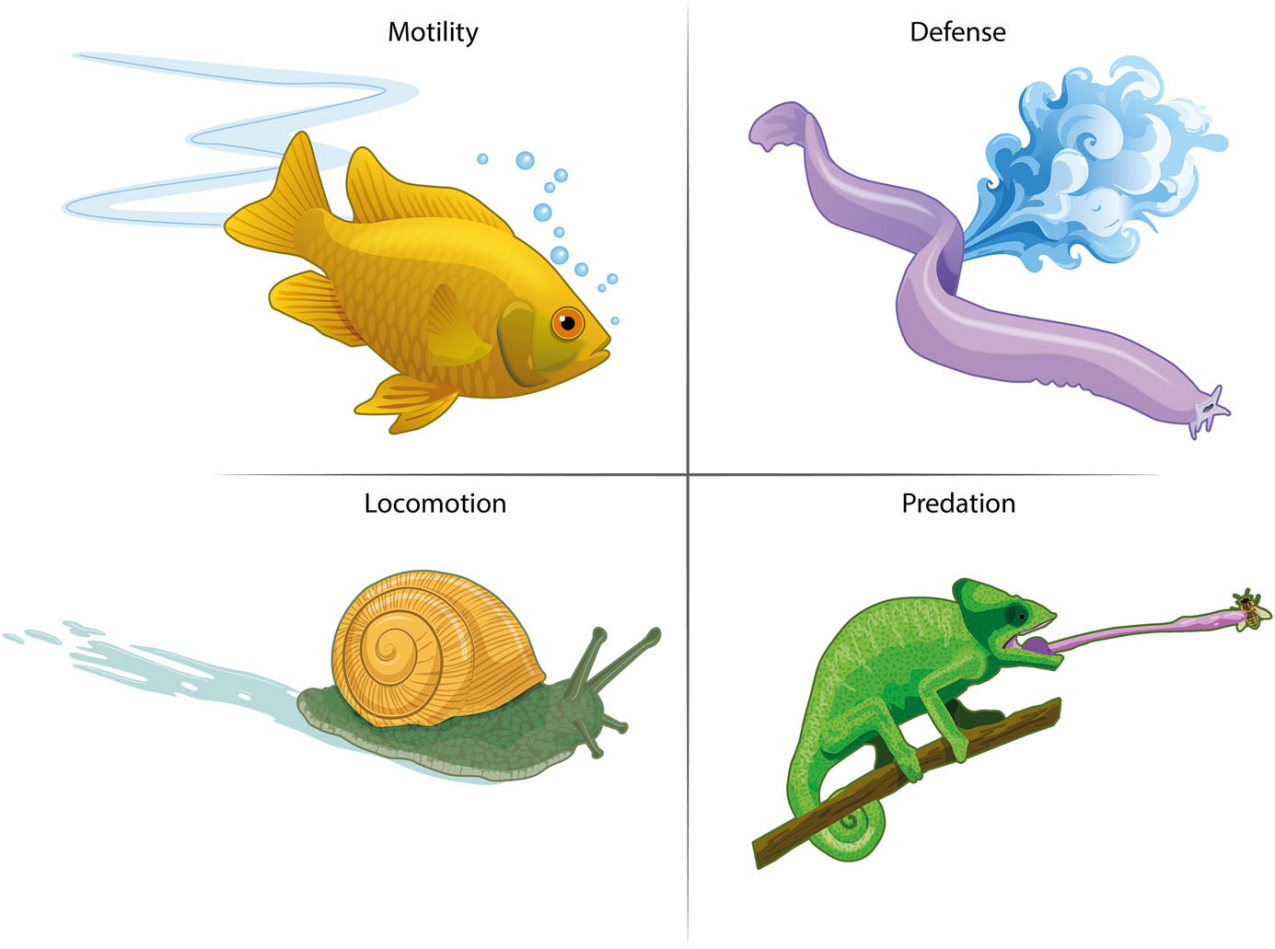
Mucus is a widespread, multifunctional secretion found throughout the animal kingdom. Mucus is not restricted to humans or even mammals but is observed across the animal kingdom, enabling an amazing diversity of functions. Shown here are examples of mucus as a medium for enhancing motility (fish, snails), as a defensive mechanism (hagfish), and as a tool for predation (chameleons). Its widespread, multifaceted nature underscores mucus as one of biology’s most versatile and evolutionarily conserved secretions.

Humans are not the only animals endowed with mucus; indeed, all mammals secrete it to protect and support their respiratory, digestive, ocular, and oral systems. Beyond mammals, virtually all animals, ranging from fish to chameleons to snails, rely on mucus to achieve a variety of unique functions (Fig. 4). For example, fish produce an external layer of mucus that not only protects against pathogens but also shields them from ultraviolet radiation and helps reduce drag when swimming. Snails and slugs leverage the viscoelastic properties of externally secreted mucus for locomotion, adhesion, and moisture retention (Liegertová and Malý, 2023). Benefitting from the stickiness of mucus, some lizards, such as chameleons, have a highly viscous mucus layer on their tongue, enabling them to more easily and securely capture their prey (Brau et al, 2016). Among the ocean’s more unusual mucus specialists are hagfish, eel-like creatures that deploy mucus as a rapid defense against predators. When threatened, a hagfish can release mucus that expands to liters within seconds, clogging the gills of attacking fish and forcing them to retreat (Fudge et al, 2003). Hagfish slime is a remarkable composite material: it contains mucins interlaced with long protein threads that uncoil in water, forming a tough yet elastic network. This combination creates a lightweight but incredibly effective deterrent that has attracted interest as a model for strong, biodegradable fibers.

“Beyond mammals, virtually all animals, ranging from fish to chameleons to snails, rely on mucus to achieve a variety of unique functions.”

From an evolutionary perspective, invertebrates such as corals, jellyfish, and sea anemones were among the first organisms to produce a functional mucus layer (Bakshani et al, 2018). Even in these simple creatures, mucus serves multiple functions. For instance, in corals, mucus layers not only form a protective barrier against physical damage and microbial infection, they can also be sloughed off to enable a self-cleaning function (Brown and Bythell, 2005). Moreover, under conditions of stress, the mucus in corals undergoes a change in composition, increasing in lipid, protein, and antibacterial activity levels to protect against infection. Viewed in this light, mucus is more than just a slimy and embarrassing secretion: it is a sophisticated, multifunctional interface between an organism and its surroundings, essential to survival in contexts as diverse as coral reefs and desert rock faces. Its ubiquity and adaptability across the animal kingdom speak to the evolutionary success of mucus as one of biology’s most versatile materials.

“…mucus is more than just a slimy and embarrassing secretion: it is a sophisticated, multifunctional interface between an organism and its surroundings, essential to survival in contexts as diverse as coral reefs and desert rock faces.”

## Mucus’s role in protecting against pollution

Because mucus plays a vital role in defending internal systems against environmental contaminants, research on mucus provides valuable information for society, supporting public health efforts to protect human health. In the lungs, mucus serves as the first line of defense against not only pathogens but also environmental pollutants such as smoke, particulate matter, and industrial chemicals. The mucus layer traps these pollutants, which are then effectively removed via mucociliary clearance. Understanding the mechanisms governing these functions can provide key insights into how a healthy mucus layer protects us from pollutants.

While healthy mucus is effective in trapping and removing external contaminants, air pollution can, in turn, impact mucus: inhaled environmental pollutants such as PM2.5 (particulate matter with a diameter of 2.5 microns or less), microplastics, and volatile organics can degrade the mucus barrier, especially in vulnerable populations exposed to high pollution levels. Recent studies have shown that, when exposed to air pollution, the respiratory mucus exhibits impaired barrier function and increased viscosity—thickened mucus that is more difficult to clear (Huff et al, 2019). Moreover, environmental pollutants can induce a weakened defense response against pathogens, with mucus becoming less effective in taming and eliminating microbial threats.

This intersection of pollution exposure and mucus barrier integrity is a rapidly emerging area of research, driven by a growing awareness of the health impacts of climate change, urbanization, and pollution. It underscores the role of mucus at the intersection of environmental and human health, highlighting the value of determining the effect of environmental particles on mucosal health to develop evidence-based standards for air quality regulation. Furthermore, because mucus-based defenses are conserved across much of the animal kingdom, pollution-driven disruption of mucosal barriers has the potential to compromise respiratory, digestive, and reproductive health in wildlife, further amplifying the ecological consequences of environmental contamination.

## Mucus as inspiration for innovation

The extraordinary properties of mucus are inspiring a new generation of biomaterials. For example, scientists are developing synthetic mucus-like coatings for wound dressings to trap pathogens and prevent infection, as well as materials that can help repair damaged native mucus layers (Bej et al, 2024). Synthetic gels that mimic the adhesive properties of mucus are promising candidates for tissue engineering and wound healing, serving as bioadhesives or scaffolds to promote tissue regeneration.

“The extraordinary properties of mucus are inspiring a new generation of biomaterials.”

By forming a lubricating coating over epithelial surfaces, mucus drastically reduces friction and prevents tissue damage in countless bodily processes, such as the smooth passage of food through the digestive tract or the effortless blinking of your eyes. Despite its importance, the mechanisms governing this lubricity have not yet been fully unraveled. Unlocking this understanding could revolutionize the development of biodegradable lubricants with wide-ranging applications from personal-care products to medical devices such as catheters or joint replacements.

The antifouling property of mucus prevents unwanted substances, including microbes and particulate matter, from sticking to surfaces, which is crucial for preventing infections and protecting against environmental pollutants. Inspired by this property, scientists are developing synthetic mucus-like coatings to prevent biofilm formation on medical implants, surgical instruments, and even the hulls of ships, significantly reducing infection risks and improving efficiency.

## A new respect for snot

Mucus, once relegated to ancient superstitions as “phlegm” and long dismissed in modern times as mere “snot”, is truly a complex and fascinating substance. Its role as a robust physical barrier, its dynamic interactions with host cells and the microbiome, and its immense potential as an inspiration for innovative biomaterials underscore its profound importance in maintaining health and driving biomedical advancement. As research expands our understanding of this often-overlooked secretion, mucus is emerging not just as a nuisance but as a blueprint for health, healing, and diagnostics, highlighting the true role of this hidden hero.

## Supplementary information

Peer Review File
