# Supplementary material for: It’s not just snot! Mucus: Nature’s Multifaceted Secretion
Source: EMBO Rep. 2026 Mar 13;27(7):1637–41. doi: 10.1038/s44319-026-00729-0 (PMC13076880; doi:10.1038/s44319-026-00729-0)
Supplement: Supplementary file 1 — Peer Review File [file 44319_2026_729_MOESM1_ESM.pdf]

EMBO reports

It's Not Just Snot! Mucus: Nature's Multifaceted Secretion

Corey Stevens and Katharina Ribbeck

Corresponding author(s): Katharina Ribbeck (ribbeck@MIT.edu)

Review Timeline:

Submission Date:	8th Dec 25
Accepted:	13th Feb 26

Editor: Holger Breithaupt

Transaction Report: This article was editorially reviewed.
